# Thermodynamic Assessment of Molten Bi_x_-Sn_1−x_ (x = 0.1 to 0.9) Alloys and Microstructural Characterization of Some Bi-Sn Solder Alloys

**DOI:** 10.3390/ma17071579

**Published:** 2024-03-29

**Authors:** Florentina Niculescu, Ion Pencea, Gheorghe Iacob, Mihai Ghiţă, Mariana-Mirela Stănescu, Mircea-Ionuţ Petrescu, Emanuel-Laurenţiu Niculescu, Mihai Buţu, Constantin-Domenic Stăncel, Nicolae Şerban, Roxana-Marina Şolea, Andrei-Alexandru Ilie

**Affiliations:** 1 Faculty of Materials Science and Engineering, National University of Science and Technology Politehnica Bucharest, 313 Splaiul Independentei, J Building, 060042 Bucharest, Romania; fniculescu@upb.ro (F.N.); ion.pencea@upb.ro (I.P.); ipetrescu@yahoo.com (M.-I.P.); emanuel.niculescu@generalsecurity.ro (E.-L.N.); mihai.butu@upb.ro (M.B.); domenic.stancel@upb.ro (C.-D.S.); nicolae.serban@upb.ro (N.Ş.); marina_roxana.solea@upb.ro (R.-M.Ş.); ilie_andrei_alexandru@yahoo.com (A.-A.I.); 2 National R&D Institute for Non-Ferrous and Rare Metals—IMNR, 102 Biruintei, 077145 Pantelimon, Romania; 3 Faculty of Applied Sciences, National University of Science and Technology Politehnica Bucharest, 313 Splaiul Independentei, J Building, 060042 Bucharest, Romania; mirela.stanescu@upb.ro

**Keywords:** Bi-Sn, solder alloys, entropy, enthalpy, free energy, optical microscopy, XRD, EDP-XRFS, SEM-EDS, characterization

## Abstract

Properties such as lower melting temperature, good tensile strength, good reliability, and well creep resistance, together with low production cost, make the system Bi-Sn an ideal candidate for fine soldering in applications such as reballing or reflow. The first objective of the work was to determine the thermodynamic quantities of Bi and Sn using the electromotive force measurement method in an electrolytic cell (Gibbs’ enthalpies of the mixture, integral molar entropies, and the integral molar excess entropies were determined) at temperatures of 600 K and 903 K. The second objective addressed is the comprehensive characterization of three alloy compositions that were selected and elaborated, namely Bi25Sn75, Bi50Sn50, and Bi75Sn25, and morphological and structural investigations were carried out on them. Optical microscopy and SEM-EDS characterization revealed significant changes in the structure of the elaborated alloys, with all phases being uniformly distributed in the Bi50Sn50 and Bi75Sn25 alloys. These observations were confirmed by XRD and EDP-XRFS analyses. Diffractometric analysis reveals the prevalence of metallic Bi and traces of Sn, the formation of the Sn_0.3_Bi_0.7_, Sn_0.95_Bi_0.05_ compounds, and SnO and SnO_2_ phases.

## 1. Introduction

The intensification of research in the field of lead-free soldering alloys started since 1 July 2006, when the new directives of the European Union (European Union Waste Electrical and Electronic Equipment Directive—WEEE and Restriction of Hazardous Substances Direction—RoHs) came into force and the European electronics industry had to be lead-free. Lead and lead compounds represent one of the 17 main categories of chemical substances and produce major problems that can threaten people’s lives or cause serious environmental imbalances [1,2].

Several conditions must be met for a soldering alloy to be appropriate: melting range that is suitable for the service temperature range; mechanical properties that are compatible with service conditions; metallurgical compatibility with surrounding metallization; low rate of intermetallic compound (IMC) formation at the service temperature; and acceptable wettability on surrounding metallization [3,4,5,6,7,8,9,10].

The demand for this category of alloys is constantly increasing due to the growth of the electronic equipment production sector, starting from applications for the common consumer (TV, PC, freezer, washing machine, mobile phone, notebook, etc.) to high-end applications such as in the field of satellite communications. Even if the replacement of traditional alloys with lead content is difficult, with their properties being difficult to match or even surpass, their drawbacks were further enhancing the properties of lead-free alloy systems by adding small amounts of alloying elements such as Bi, Cu, In, Ag, Al, Ga, Sb, Cr, Ni, and Ge to develop ternary and even quaternary Pb free systems [11,12,13,14,15].

Newly developed lead-free alloy systems have been discovered that can successfully replace them [16], such as those in the binary Sn-Cu, Sn-Ag, Sn-Bi, Sn-Zn, Sn-In, and Sn-Sb systems or the ternary Sn-Zn-Bi and Sn-Ag-Cu systems (SAC solders), with acceptable properties (melting temperature, mechanical properties, microstructure, wettability, solder ability, reliability, and cost), thus offering a positive trend in terms of finding new variants of accessible lead-free soldering alloys with properties similar to those prohibited [6,7,8,9,10,11,13]. Other investigated alloys are from the Bi-Ag, Bi-Sb, Bi-Sn, Bi-Zn, or Bi-Sn-Zn systems, where further investigations are necessary for better understanding. The addition of Bi decreases the melting point of the system and improves the wettability, and up to a certain extent, the mechanical properties could also be improved [7,17,18,19,20].

In both the conventional electronic industry (the industry that produces stand-alone equipment) and industrial electronics (equipment and components incorporated into products), the Bi-Sn alloy system is frequently used worldwide. The electronic industry is a major consumer of special materials such as semiconductors, insulators, and metals used in the construction of various electronic devices.

According to current research practice and the international research approach, the trend in materials science is a fundamental comprehensive approach to understanding material properties as accurately as possible, especially the physico-chemical characteristics essential for the intended applications. For this reason, we are increasingly moving toward a fundamental approach to material properties based on fine structure in correlation with other influencing factors that contribute to the measured values of those properties. In this regard, techniques and methods for investigating material structures at the atomic level promote exceptional scientific results, leading to the development of advanced materials for all practical areas of interest (e.g., metallurgy, medicine, electronics, etc.) [21,22,23,24,25,26,27,28,29,30]. The phenomenology associated with quantities such as Gibbs’ enthalpy, thermodynamic activity, entropy, free enthalpy, etc., must be seen from the perspective of material structure (phase stability analysis or phase transformations of an alloy system). These data are essential because they can provide us with valuable information, even if, in most cases, they are based on empirical calculation methods and on the problems of developing alloy systems or refining them, while knowing what problems face the development of the category of alloys for soldering.

The novelty of this research is the performance of detailed calculations regarding certain thermodynamic functions based on new research carried out on alloys from the Bi-Sn system by reviewing the data at an established temperature (600 K) as well as at a temperature at which no studies have been carried out until now (903 K). Another element of novelty is the development and study of new compositions of Bi-Sn alloys for solders (Bi25Sn75, Bi50Sn50, and Bi25Sn75) and the performance of detailed investigations on the micro-structural changes that occurred following an increase in the content of bismuth in the alloy mass (the appearance of phases and compounds that are not quite studied in the specialized literature regarding these types of alloys).

## 2. Materials and Methods

### 2.1. Elaboration of the Bi-Sn Solder Alloys

**Figure 1:**
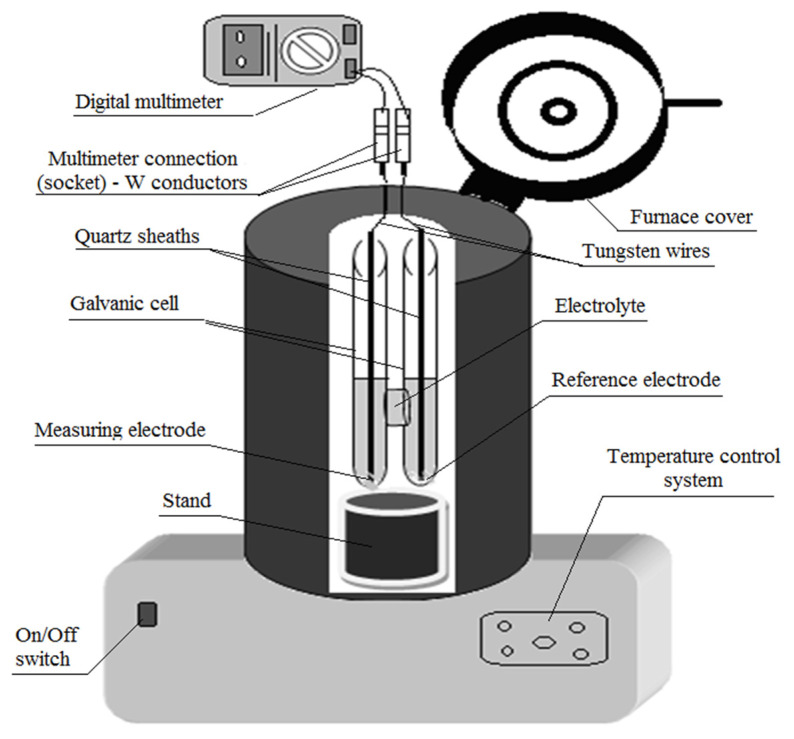
The drawing of the installation used in the experiments [7].

The elaboration of soldering alloys was carried out in an electric induction furnace, with a capacity of 2 kg, equipped with a PID temperature controller 220 V model REX-C100 (RKC Instruments Inc., Tokyo, Japan), which has an accuracy of ±0.5% FS ±1 °C (274.15 K). The maximum temperature reached by the furnace is 1100 °C (1373 K), and its schematic presentation is shown in Figure 1.

Bi, with a purity of 99.6%, and Sn, with a purity of 99.8%, were used to prepare the Sn-Bi alloys. Sn and Bi primary were introduced in the preheated crucible. The furnace (S Y Electric Melting Furnace, Italy) temperature was set to 903 K, and it reached the temperature of 903 K in approximately 25 min. The elaboration process proceeded with the preparation of materials and raw materials, encompassing both primary preparation and charging preparation stages.

During primary preparation, a thorough assessment of the raw materials and auxiliary materials was conducted for qualitative and quantitative verification. Additionally, within this stage, the raw materials were dosed, sized, and weighed using an electronic balance. The obtained samples (Bi25Sn75, Bi50Sn50, and Bi75Sn25) underwent a process of melting and casting within graphite crucibles and molds. 

The melting temperature was maintained constant for 10 min and subjected to a refining operation with 0.5% flux (3 portions), immersed in the molten bath with a graphite rod, and subsequently homogenized before pouring into the graphite mold. The alloy was cleaned of the slag formed and poured into the mold at a temperature of 623 K using a crucible handling tong. Refined ammonium chloride (NH_4_Cl) served as a protective flux to shield the molten metal from oxidizing gases within the furnace environment.

### 2.2. Determination of Thermodynamic Functions of the Bi-Sn System

The aim of this research was to obtain extensive knowledge about the thermodynamic behavior of Bi-Sn alloys in the molten state over a large temperature; the selection of the values of 600 K and 903 K has a certain motivation.

First, tests were carried out at a temperature of 600 K to verify and correlate the thermodynamic data with those obtained by Magnus and Mannheimer [31] and, at the same time, to verify the correctness of the measurement process using the electromotive force method (EMF) which ensures greater precision and accuracy of the results.

Secondly, tests were carried out to determine the thermodynamic functions at a temperature of 903 K since there are no specialized studies carried out by other researchers so far, thus representing a challenge for the team. 

The thermodynamic properties of the Bi-Sn system were also investigated at temperatures of 673 K, 723 K, and 793 K using EMF measurements [23,32]. Another study on the Bi-Sn binary system concerns the measurement of the integral enthalpy of the mixture carried out at temperatures of 767 K and 855 K by calorimetric methods [33] or vapor pressure at a temperature of 1373 K [34].

Bi-Sn solder alloys (Sn-5Bi, Sn-15Bi, Sn-30Bi, Sn-45Bi, and Sn-58Bi) were elaborated in a ceramic crucible at a temperature of 873 K, cooled to 573 K and then chilled and cast in a graphite mold and was observed that the Bi addition modify the microstructure, interfacial behaviors, and joint strength [35]. 

Melting of Bi-Sn alloys to 903 K can offer several advantages in various applications: improved thermal resistance (advantageous in applications where parts must withstand high temperatures), dimensional stability (not to deform or contract excessively during cooling and use), improved mechanical performance (corrosion resistance or durability), ease of processing (easier to machine and form into various shapes and configurations), and versatility in demanding applications (where a reliable and durable material is required). For electronics applications, Bi-Sn alloys can provide good adhesion between electronic components and the circuit board without affecting their operation.

The tests to determine the thermodynamic functions were carried out using the same furnace mentioned previously, which was used for the elaboration of the alloys, in order not to influence the results. At the same time, the use of this furnace allowed the introduction and fixing of an H-shaped electrochemical cell used for thermodynamic determinations (vessel height—15 cm; cylinder inner diameter—1.8 cm; the thickness of quartz walls –0.2 cm; the length of the W wires—20 cm; connection channel diameter—1.8 cm; and the height of the connecting channel—2.5 cm). 

Following the cell preparation, i.e., introducing the pure Bi electrode, Bi-Sn electrode, and the electrolyte (a mixture of 17% moles KCl and 83% moles SnCl_2_) into the cell compartments, the cell was placed into the furnace and slowly heated until the furnace had reached the envisaged temperatures, namely 600 K and 903K. Once the working temperature was stabilized, the W conductors were introduced into the working cell, put into contact with the melted electrodes, and coupled to the digital multimeter [7,22]. 

The electrochemical system was maintained at a constant temperature until the cell’s electromotive voltage became constant, which is considered the equilibrium electromotive voltage (*E*) of the cell. The equilibrium value of the electromotive voltage was reached after approximately 20 min, and five measurements were carried out on each molar fraction in the concentration range [0.1 to 1.0] [7].

Based on the measured values, we were able to calculate the thermodynamic activity as well as the thermodynamic activity coefficients for Bi [7,22]. Using the calculated values for Bi depending on concentration, the activity of the Sn component (as_n_) could be determined at constant temperature based on the Gibbs-Duhem relationship [36,37]. 

After obtaining these thermodynamic functions, were calculated further the partial molar enthalpy of the mixture ($∆{\bar{H}}_{i}$), partial molar entropies of the mixture (($∆{\bar{S}}_{i}$), integral molar free energies ($∆G_{i}$) and the integral molar excess energies ($∆G_{i}^{E}$), integral molar enthalpies ($∆H_{i}$) and integral molar excess enthalpies ($∆H_{i}^{E}$) and integral molar entropies ($∆S_{i}$) and the integral molar excess entropies ($∆S_{i}^{E}$). The obtained results were processed using the Mathcad 15 analysis software and transposed in Excel for a better graphic display.

### 2.3. Sample Characterization

Conventional cast samples of binary Bi-Sn alloys underwent characterization through optical microscopy using the Optika model B383 MET optical microscope, (Optika, Ponteranica, Italy) which was equipped with a digital camera and image processing software. Prior to microscopic examination, the samples underwent metallographic preparation to reveal their structural properties. The resulting micrographs depicted variations in the microstructure corresponding to the percentage composition of bismuth within each sample.

The microstructural characterization was conducted through scanning electron microscopy (SEM) FEI QUANTA 250 (FEI Company, Hillsboro, OR, USA), in high vacuum mode, backscattered electron techniques (BSE) using an Angular Backscattered Detector. Point analysis, elemental mapping, and semi-quantitative analysis were performed through energy dispersive X-ray spectroscopy (EDS) using the EDAX Element EDS Analysis System consisting of ELEMENT Silicon Drift Detector Fixed and ELEMENT EDS Analysis Software Suite (EDAX APEX™, DigitalMicrograph 3.6). For microstructural investigations, metallographic specimens were prepared by sectioning, mounting, grinding, fine grinding, polishing, and etching with a H_2_O_2_ (30%):H_2_O mixture.

For comprehensive elemental and structural characterization of Bi-Sn alloys, X-ray fluorescence spectrometry (XRFS) and X-ray diffractometry (XRD) were identified as the most effective analytical approaches. These instrumental techniques require minimal sample preparation while providing extensive information on elemental and phase compositions. The Energy Dispersive Polarization X-ray Fluorescence (EDP-XRFS) method utilized for the characterization of Bi-Sn alloys employed the XEPOS spectrometer (SPECTRO Analytical GmbH). The obtained spectra were processed using the analytical program TurboQuant-Alloys.

X-ray diffraction investigations were conducted using the Bruker D8 Advance X-ray diffractometer in a Bragg-Brentano configuration for the interval 2θ = 10…90° (Δ_2θ_ = 0.02°, λ_Cu_ = 1.5415 Å). The structural data required for comparison were taken from the ICDD (International Center for Diffraction Data), formally JCPDS—(Joint Committee on Powder Diffraction Standards) files. The identification of the nature of the phase was performed by considering the positioning of the diffraction lines, respectively, the interplanar distances *d*_*h k l*_ associated with the diffraction maxima through the Bragg relation. Based on the intensities of the diffraction lines, the volume concentration of the phases in the analyzed sample is established in a semi-quantitative way, conducted using the Bruker-EVA program version 4.2.1.10, and was indexed based on the ICDD-PDF4+ database.

## 3. Results and Discussion

### 3.1. Thermodynamical Assessment

After calculating the activity coefficients, the partial molar enthalpy of the mixture was calculated based on the following relationship:

(1) $$∆{\bar{H}}_{i}\;=\;∆{\bar{G}}_{i}+T∆{\bar{S}}_{i},$$

where

$∆{\bar{H}}_{i}$—the partial molar enthalpy of the mixture for element *i* [J/mol];

$∆{\bar{G}}_{i}$—the partial molar free enthalpy of the mixture for element *i* [J/mol];

$∆{\bar{S}}_{i}$—the partial-molar entropy of the mixture for element *i* [J/mol].

*T*—temperature, [K].

- The values of the partial molar enthalpies of the mixture ($∆{\bar{H}}_{i}$) of Bi and Sn at 600 K and 903 K, calculated based on Equation (1), are presented in Table 1.

- The calculation of the values of partial molar entropies for the mixture was performed based on the following relationship (2):

(2) $$∆\bar{S_{i}}=-\frac{\partial ∆\bar{G_{i}}}{\partial T}=-Rlnx_{i}-\frac{\partial }{\partial T}\left(RTlny_{i}\right)=-Rlnx_{i}-\frac{\partial ∆{\bar{G}}_{i}^{E}}{\partial T}$$

where

$∆\bar{S_{i}}$—the partial molar entropy of the mixture for element *i* [J/mol];

$∆{\bar{G}}_{i}$—the partial molar free enthalpy of the mixture for element *i* [J/mol];

$∆{\bar{G}}_{i}^{E}$—the partial molar excess free enthalpy of the mixture for element *i* [J/mol];

*R*—the universal gas constant [J/mol·K];

*T*—temperature, [K].

The partial molar entropies of the mixture ($∆{\bar{S}}_{i}$) for Bi and Sn, calculated using Equation (2), are presented in Table 2.

- The integral molar free energies ($∆G_{i}$) and the integral molar excess energies ($∆G_{i}^{E}$) at 600K and 903K have been calculated using Equations (3) and (4), and they are presented in Table 3.

(3) $$\Delta G_{i}=\sum x_{i}\Delta {\bar{G}}_{i}=x_{Bi}\Delta {\bar{G}}_{Bi}+x_{Sn}\Delta {\bar{G}}_{Sn}$$

(4) $$\Delta G^{E}=\Delta G-\Delta G^{id}=RT(x_{Bi}ln\gamma_{Bi}+x_{Sn}ln\gamma_{Sn})$$

where

$\Delta G_{i}$—the molar-free enthalpy of the mixture for element *i* [J/mol];

$∆{\bar{G}}_{i}$—the partial molar free enthalpy of the mixture for element *i* [J/mol];

$\Delta G^{E}$—the integral molar excess free enthalpy of the mixture for element *i* [J/mol];

ΔG—free energy variation [J/mol];

$\Delta G^{id}$—ideal free energy variation [J/mol];

*R*—the universal gas constant [J/mol·K];

*T*—temperature, [K].

**Table 3:** The integral molar free and the integral molar excess energies at T = 600 K and T = 903 K.

	Temperature 600 K		Temperature 903 K	
*X*_*Bi*_	∆G [J/mol]	$∆G^{E}$ [J/mol]	∆G [J/mol]	$∆G^{E}$ [J/mol]
0.0	0	0	0	0
0.1	−6425	−35,620	−602	−9820
0.2	−5802	−32,060	−1177	−11,850
0.3	−5292	−28,590	−1739	−14,450
0.4	−4906	−21,990	−2249	−17,400
0.5	−4679	−18,920	−2853	−20,550
0.6	−4672	−16,060	−3593	−23,830
0.7	−4946	−13,490	−4697	−27,180
0.8	−5674	−11,370	−6548	−30,570
0.9	−7157	−10,000	−10,530	−33,970
1.0	0	0	0	0

- The integral molar enthalpies ($∆H_{i}$) and integral molar excess enthalpies ($∆H_{i}^{E}$) at temperatures of 600 K and 903 K were calculated using Equations (5) and (6) and are presented in Table 4.

(5) $$\Delta H_{i}=\sum x_{i}\Delta {\bar{H}}_{i}=x_{Bi}\Delta {\bar{H}}_{Bi}+x_{Sn}\Delta {\bar{H}}_{Sn}$$

(6) $$∆H^{E}=∆H^{M}-∆H^{id}=∆H^{M}$$

where

$\Delta H_{i}$—the integral molar enthalpies of the mixture [J/mol];

$\Delta {\bar{H}}_{i}$—the partial molar enthalpies of the mixture [J/mol];

$∆H^{E}$—the variation excess enthalpy [J/mol];

$∆H^{M}$—the variation of the integral molar enthalpy [J/mol];

$∆H^{id}$—ideal free enthalpy variation [J/mol].

**Table 4:** The integral molar enthalpies and integral molar excess enthalpies of Bi and Sn at T = 600 K and T = 903 K.

	Temperature 600 K		Temperature 903 K	
*X*_*Bi*_	∆H [J/mol]	$∆H^{E}$ [J/mol]	∆H [J/mol]	$∆H^{E}$ [J/mol]
0.0	0	0	0	0
0.1	−19,960	−18,140	−13,050	−17,130
0.2	−11,360	−14,020	−8486	−11,370
0.3	−8794	−11,410	−5593	−8019
0.4	−6838	−9188	−3628	−5280
0.5	−5248	−7031	−2278	−2672
0.6	−3908	−4865	−1342	−95
0.7	−2769	−2791	−698	2391
0.8	−1756	−870	−295	4616
0.9	−817	736	−68	6381
1.0	0	0	0	0

- The integral molar entropies ($∆S_{i}$) and the integral molar excess entropies ($∆S_{i}^{E}$) of the alloy Bi–Sn at temperatures of 600 K and 903 K, they were calculated using Equations (7) and (8) and are presented in Table 5.

(7) $$\Delta S_{i}=\sum x_{i}\Delta {\bar{S}}_{i}=x_{Bi}\Delta {\bar{S}}_{Bi}+x_{Sn}\Delta {\bar{S}}_{Sn}$$

(8) $$\Delta S^{E}=\Delta S-\Delta S^{id}=\frac{\Delta H^{E}-\Delta G^{E}}{T}+R(x_{Bi}lnx_{Bi}+x_{Sn}lnx_{Sn})=\frac{\Delta H^{E}-\Delta G^{E}}{T}$$

where

$\Delta S_{i}$—integral molar entropies of the mixture for element *i* [J/mol];

$∆\bar{S_{i}}$—the partial molar entropy of the mixture for element *i* [J/mol];

$\Delta S_{i}^{E}$—integral excess molar entropy of the mixture for element *i* [J/mol];

$∆H^{E}$—the variation excess enthalpy [J/mol];

$\Delta G^{E}$—the integral molar excess free enthalpy of the mixture for element *i* [J/mol];

*R*—the universal gas constant [J/mol·K];

*T*—temperature, [K].

**Table 5:** The integral molar entropies and the integral molar excess entropies at T = 600 K and T = 903 K.

	Temperature 600 K		Temperature 903 K	
*X*_*Bi*_	∆S [J/mol]	$∆S^{E}$ [J/mol]	∆S [J/mol]	$∆S^{E}$ [J/mol]
0.0	0	0	0	0
0.1	−1.189	1.514	−1.175	1.484
0.2	0.922	5.082	0.823	4.784
0.3	3.850	8.929	2.954	7.726
0.4	6.186	11.781	5.881	10.690
0.5	7.469	13.232	6.841	12.467
0.6	7.632	13.228	6.326	12.563
0.7	6.726	11.805	5.765	10.679
0.8	4.890	9.051	3.909	8.611
0.9	2.606	5.309	1.806	4.629
1.0	0	0	0	0

### 3.2. Characterization of Bi-Sn Solder Alloys

#### 3.2.1. Optical Microscopy

Due to the slow cooling of the samples, micro-cracks have been formed, and the grain size is large. This aspect can be improved through rapid or forced cooling of the samples and, of course, achieving a finer structure if the alloy is intended for use in “chip-off/chip-on” or “re-balling” techniques for repairing processors or memory devices in electronics. However, these techniques represent a small percentage of the electronics market.

**Figure 2:**
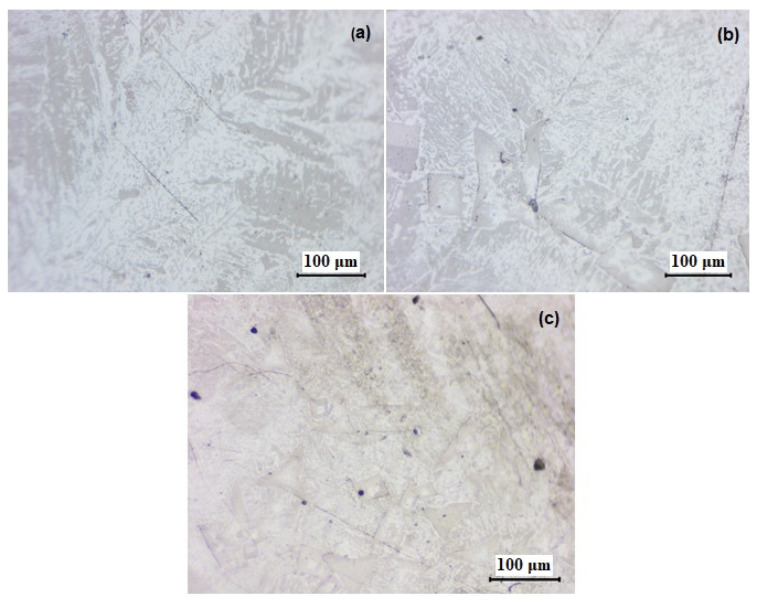
The optical microstructures for the following alloy samples (magnification 50×): (**a**) Bi25Sn75; (**b**) Bi50Sn50; (**c**) Bi75Sn25.

According to the resulting microstructures, significant changes in the structure of the elaborated alloys are observed (Figure 2a–c). From a structure primarily based on the Sn matrix with few areas where rare zones with intermetallic constituents have been formed, we have transitioned to a much more balanced structure in this regard. This new structure exhibits a greater quantity of intermetallic constituents, metallic phases, and even the formation of compounds with polyhedral or tetragonal shapes.

In the microstructures, we can observe the α phase, which is rich in bismuth (gray areas), and the β phase, which is rich in tin (white areas). No voids or discontinuities are visible in the analyzed section. This is because, unlike other metals, Bi expands upon solidification. The presence of these two phases indicates significant diffusion during the cooling of the melt.

According to the phase diagram of Bi-Sn [19], the solubility limit of Bi in Sn is approximately 6 at. % for a temperature of 138 °C. Considering that the percentages of Bi used in the alloy compositions during fabrication greatly exceed this value, bismuth is observed to precipitate from the tin-rich matrix, resulting in the observed structures.

All phases are uniformly distributed in the Bi75Sn25 alloy structure without the formation of conglomerates and/or clusters that would cause heterogeneity in chemical composition. Instead, a fine and uniformly distributed multiphase structure is evident. Fine grains of Bi, some of them with rounded shapes, can be observed to have precipitated within the Sn matrix.

These observations are confirmed by XRD, ED-XRFS, and SEM-EDS investigations.

#### 3.2.2. SEM-EDS Investigations

To highlight the effect of bismuth on the microstructure of Bi-Sn alloys (Bi25Sn75, Bi50Sn50, and Bi75Sn25), scanning electron microscopy (SEM) investigations coupled with energy dispersive spectrometry (EDS) micro-compositional analyzes were carried out.

**Figure 3:**
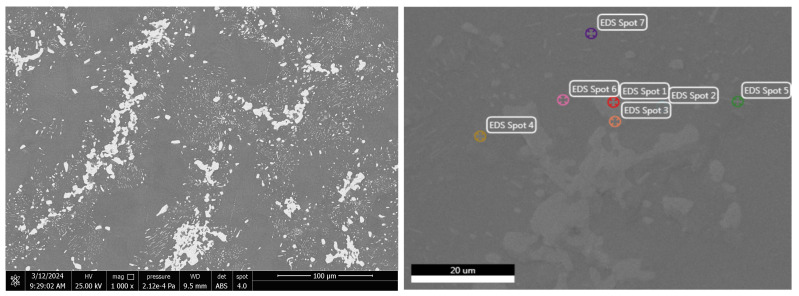
SEM images on the microstructure of Bi25Sn75 alloy sample.

**Figure 4:**
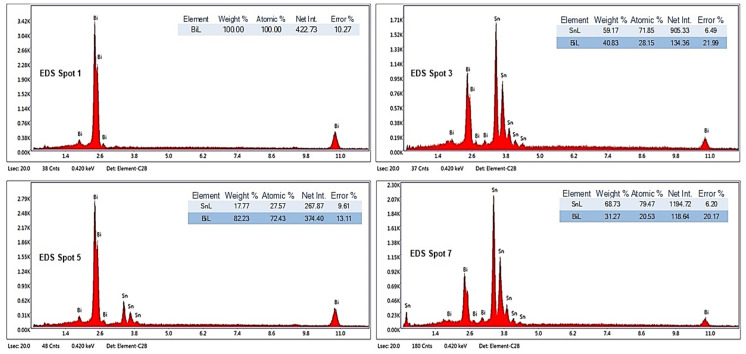
EDS analysis in different spots of Bi25Sn75 alloy sample.

**Figure 5:**
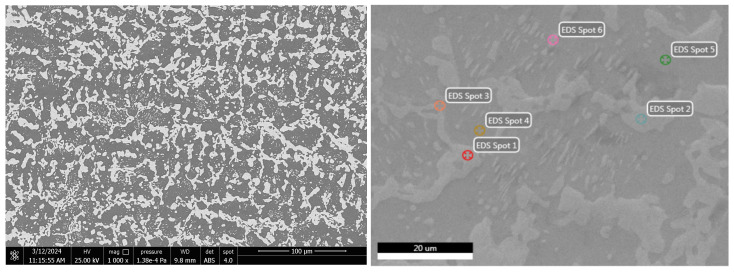
SEM images on the microstructure of Bi50Sn50 alloy sample.

**Figure 6:**
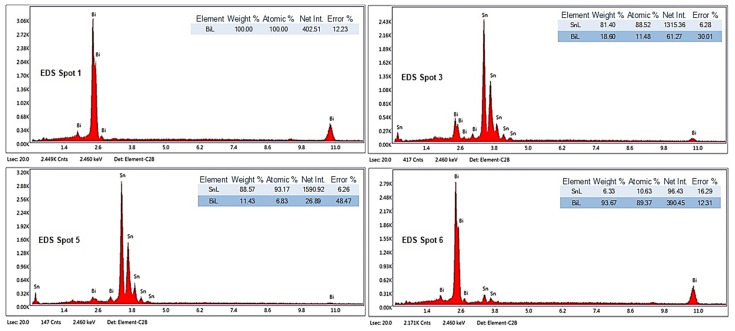
EDS analysis in different spots of Bi50Sn50 alloy sample.

**Figure 7:**
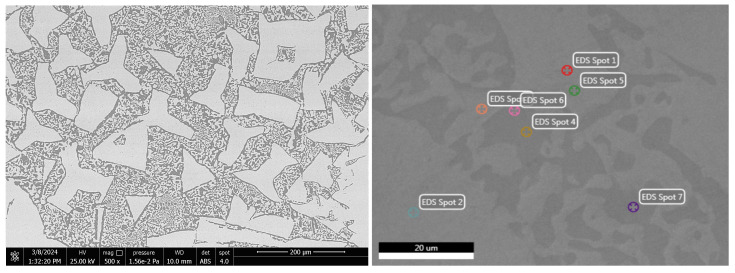
SEM images on the microstructure of Bi75Sn25 alloy sample.

**Figure 8:**
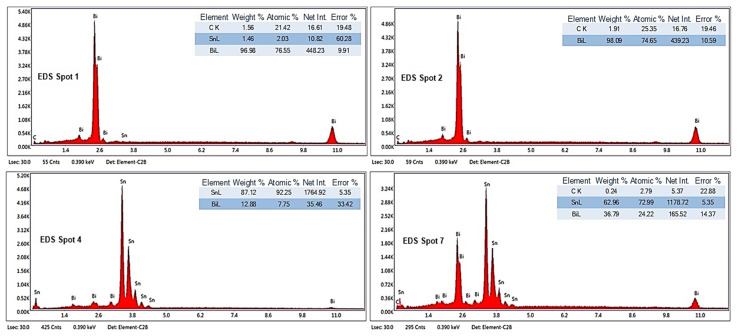
EDS analysis in different spots of Bi75Sn25 alloy sample.

Analyzing the SEM-EDS images in Figure 3, Figure 4, Figure 5, Figure 6, Figure 7 and Figure 8, it is found that Bi has an obvious effect on the formation of the alloy structure and implicitly on the formation of phases and metallographic constituents. Bismuth distribution is homogeneous in all analyzed microstructures. In all studied alloys, Sn-Bi or Bi-Sn solid solutions were formed, composed of β-Sn phases in which fine Bi particles are embedded.

Several EDS analyses were carried out (Figure 4, Figure 6 and Figure 8) at the points marked on the microstructures to determine the micro-composition of the analyzed regions. They are presented together with the SEM microstructures.

Following the SEM-EDS analysis, the microstructural transformations in the studied alloys are quite visible. The darker areas represent b-Sn primary phases, and the lighter areas are eutectic structures between Sn-Bi and Bi-Sn. By increasing the bismuth content, the eutectic structure increases and the b-Sn phase decreases. The Bi25Sn75 alloy shows a typical hypoeutectic structure, with areas rich in bismuth; by equalizing the metal content, the Bi50Sn50 alloy shows a eutectic structure with bismuth spread homogeneously throughout the mass of the alloy, and in the last alloy, Bi75Sn25, by increasing the bismuth content, it is created a typical hypereutectic structure, with large bismuth grains between which different Bi-Sn phases are formed.

Also, from the microstructures in Figure 5 (Bi50Sn50 alloy) and Figure 7 (Bi75Sn25 alloy), the formation of fine needle-shaped beta phases embedded in the alpha phase dendritic matrix can be observed.

#### 3.2.3. Structural Analysis through XRD and ED-XRFS

**Figure 9:**
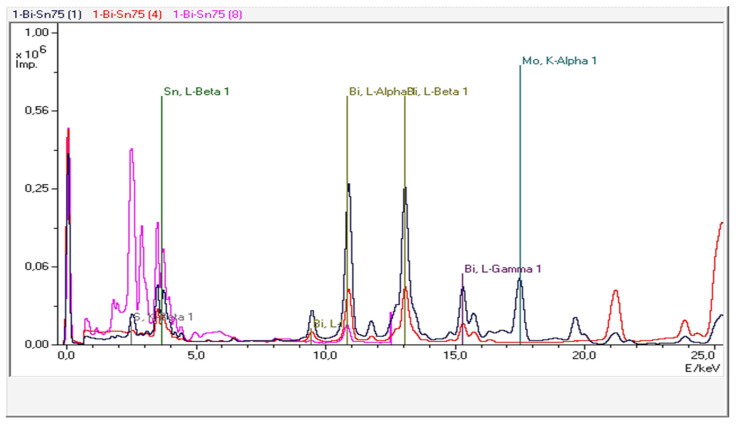
The EDP-XRF spectra correspond to the Bi25Sn75 alloy sample.

Three representative alloys were investigated, namely Bi25Sn75, Bi50Sn50, and Bi75Sn25. The Bi-Sn samples provided three XRF spectra (Figure 9, Figures 11 and 13) obtained through the secondary scattering of Rh X-ray radiation on Al_2_O_3_ targets—the blue curve; Mo targets—the red curve; and HOPG targets—the magenta curve.

It can be observed that the fluorescence lines of Sn and Bi are dominant. Additionally, the XRF spectra also exhibit accompanying lines due to Compton scattering on secondary targets. The elemental concentrations provided by the TurboQuant-Alloys analytical program are presented in Table 6, Table 7 and Table 8, in which the concentrations of the main elements—Bi and Sn can be observed with values very close to those calculated for loading in the furnace. The other elements that appear come from the materials used, which do not have a very high degree of purity. Most of the values represent negligible amounts.

**Figure 10:**
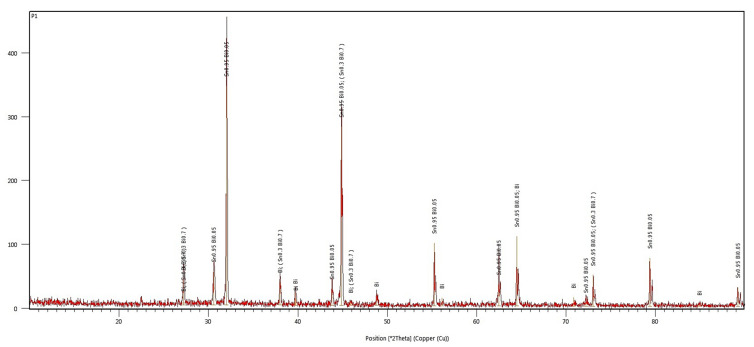
The diffractogram of the Bi25Sn75 alloy.

The diffractogram from Figure 10 shows the diffraction peaks of the Bi metal, Sn_0.3_Bi_0.7_, and Sn_0.95_Bi_0.05_ compounds in the obtained Bi25Sn75 alloy. This demonstrates a strong interaction between metals, leading to a precipitation of intermetallic compounds in the β Sn matrix (according to EDP-XRF spectra from Figure 9), creating a rather solid solution of Bi in Sn.

The Sn_0.95_Bi_0.05_ compound is mentioned in a paper of [38] where Bi-added PbSn solders were used as joint samples fabricated from NbTi and Bi2223 wires using an in-situ sheath-dissolution method. In the solder alloy, a major phase (Pb_0.63_Sn_0.1_Bi_0.27_) and a minor phase (Sn_0.95_Bi_0.05_) were shown. According to the authors, this phase is identified as small islands that do not show superconductivity at liquid He temperature.

The semi-quantitative phase analysis reveals that the sample consists of two phases rich in Sn, accounting for approximately 90% of Sn and 9% of segregated Bi. The 25% of Bi is found in two states—diffused and segregated—within Sn, indicating the potential formation of a Bi-Sn eutectic.

**Figure 11:**
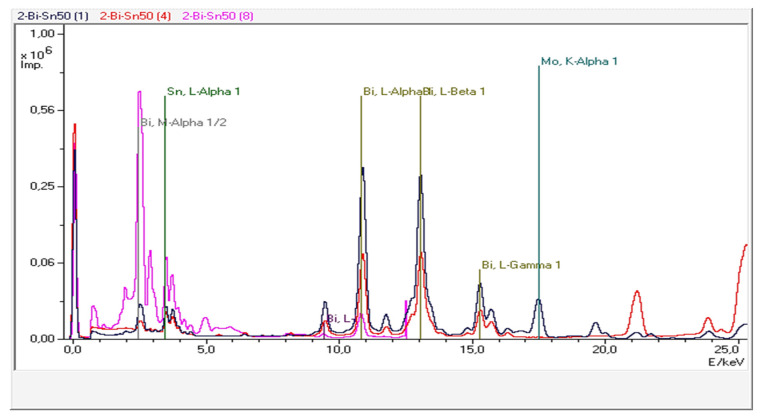
The EDP-XRF spectra correspond to the Bi50Sn50 alloy sample.

The EDP-XRFS test conducted on the Bi50Sn50 alloy sample provided fluorescence spectra with more intense lines in the low-energy region of the spectra due to the increased proportion of Bi in the alloy (Figure 11).

**Figure 12:**
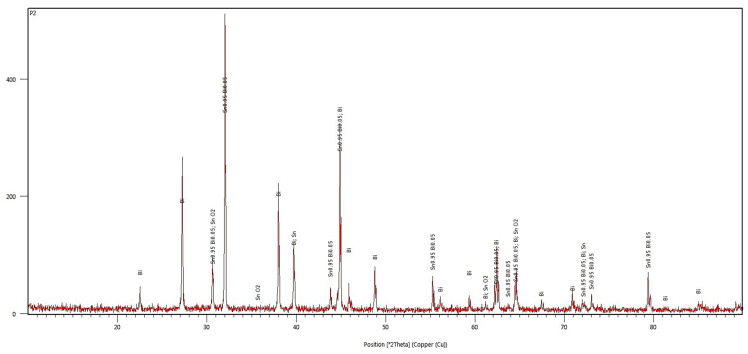
The diffractogram of the Bi50Sn50 alloy.

The diffractometric investigations carried out on the Bi50Sn50 alloy sample reveal an increase in the intensity of the diffraction lines associated with the metallic Bi phase (Figure 12) and a decrease in the intensity of the lines of the Sn_0.95_Bi_0.05_ compound. In the diffractogram, it is evident that the Sn_0.3_Bi_0.7_ compound no longer appears, but the orthorhombic SnO_2_ phase (cassiterite) is observed.

The diffractogram in Figure 12 does not show lines from phases other than those mentioned earlier, indicating that the alloy does not contain crystallized impurities.

**Figure 13:**
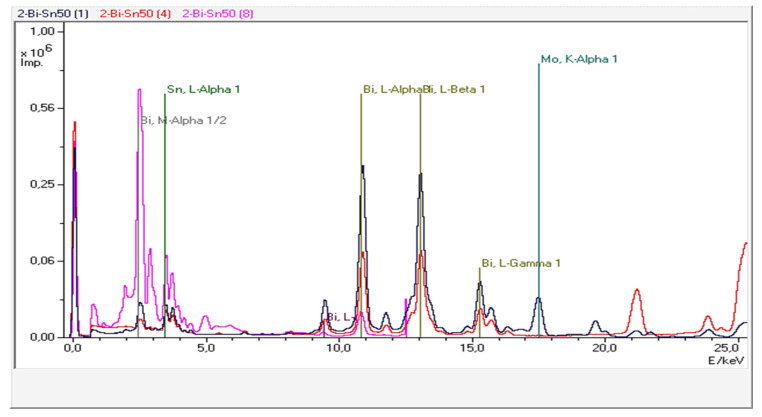
The EDP-XRF spectra correspond to the Bi75Sn25 alloy sample.

The XRF spectra for the Bi75-Sn25 alloy sample (Figure 13), obtained in the same manner as the previous spectra, exhibit intense characteristic spectral lines, which facilitated a highly accurate elemental analysis, as shown in Table 8.

**Figure 14:**
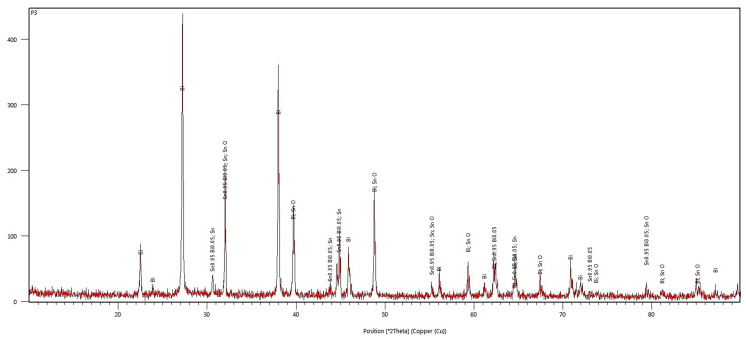
The diffractogram of the Bi75Sn25 alloy.

The diffractometric analysis confirms the presence of metallic Bi in the Bi75Sn25 alloy sample (Figure 14), the formation of the Sn_0.95_Bi_0.05_ compound, along with traces of metallic Sn. The orthorhombic SnO_2_ (cassiterite) phase no longer appears; it was replaced by the tetragonal SnO phase. This phase transformation occurs at temperatures above 573 K. These tin oxides are part of a class of materials with high electrical conductivity, being of interest in many fields, but the most important ones are electronics or optoelectronics.

The elemental composition of the Bi75-Sn25 alloy sample closely aligns with the projected composition of the standard. Residual elements identified in the sample exhibit values near the detection limit of the spectrometer. The origin of characteristic spectral lines attributed to these residual elements may stem from impurities present in the precursors or artifacts like the Compton scattering of specific Sn and Bi fluorescence photons.

## 4. Conclusions

According to the first objective of the paper, we successfully obtained temperature-dependent thermodynamic properties—enthalpy, entropy, and Gibbs free energy—for Sn and Bi at temperatures of 600 K and 903 K. The obtained experimental values revealed consistent trends, demonstrating agreement between the obtained and expected values. This confirms the accuracy of the experimental procedure. The derived thermodynamic functions enable precise calculations at any temperature–concentration point of interest with high accuracy.

According to the second objective of the work, three types of alloys, namely Bi25-Sn75, Bi50-Sn50, and Bi75-Sn25 were cast and investigated using optical microscopy, SEM-EDS, XRD, and EDP-XRFS methods.

- Microstructural variations were observed depending on the bismuth content, with structures exhibiting Sn-based matrices, Bi-based matrices, and equilibrated structures with numerous intermetallic constituents. Bismuth has an obvious effect on the formation of the alloy structure. The absence of conglomerates or clusters causing heterogeneity in chemical composition was noted. Metallic phases were uniformly distributed, and the formation of polyhedral, tetragonal, or orthorhombic compounds was observed.
- The SEM-EDS characterization revealed significant changes in the structure of the elaborated alloys. It starts with a hypoeutectic structure (Bi25Sn75), and as the bismuth content is increased, a hypereutectic structure (Bi75Sn25) is reached. The formation of fine needle-shaped beta phases embedded in the alpha phase dendritic matrix is highlighted for the Bi75Sn25 alloy.
- The EDP-XRFS analyses revealed that the concentrations of the main elements, Bi and Sn, can be observed with values that are very close to those calculated, as well as the presence of negligible amounts of other elements. Sample one exhibited dominant fluorescence lines of bismuth and tin, with semi-quantitative phase analysis indicating the presence of two Sn-rich phases and segregated bismuth. Sample two showed more intense fluorescence lines in the low-energy region due to increased bismuth content, with diffractometric investigations confirming the intended alloy state. Sample three closely matched the projected standard composition, with identified residual elements attributed to impurities or artifacts.
- Diffractometric analysis revealed the prevalence of metallic bismuth and the formation of the Sn_0.3_Bi_0.7_ and Sn_0.95_Bi_0.05_ compounds, along with traces of metallic tin, thus demonstrating strong interactions between Bi and Sn leading to the formation of different solid solutions. The presence of SnO is undoubted, as its diffraction lines overlap with the lines of other indexed phases (Figure 14). Also, the lines of SnO_2_ overlap except for a line of very small intensity (Figure 13).

Further research will be carried out to establish the optimal Bi-Sn alloy for soldering purposes, including mechanical tests, melting temperature variation, and wetting properties depending on circuit board characteristics.

## Figures and Tables

**Table 1:** Partial molar enthalpies of Bi and Sn la T = 600 K and T = 903 K.

	Temperature 600 K		Temperature 903 K	
*X*_*Bi*_	$∆{\bar{H}}_{Bi}$ [J/mol]	$∆{\bar{H}}_{Sn}$ [J/mol]	$∆{\bar{H}}_{Bi}$ [J/mol]	$∆{\bar{H}}_{Sn}$ [J/mol]
0.0	−6.093 × 10^3^	−5.812 × 10^3^	−9.166 × 10^3^	−4.054 × 10^3^
0.1	−3.389 × 10^3^	−2.593 × 10^3^	−5.100 × 10^3^	0.302 × 10^3^
0.2	−1.188 × 10^3^	−1.188 × 10^3^	−1.788 × 10^3^	3.674 × 10^3^
0.3	0.498 × 10^3^	0.117 × 10^3^	0.751 × 10^3^	6.533 × 10^3^
0.4	1.978 × 10^3^	2.270 × 10^3^	2.977 × 10^3^	9.077 × 10^3^
0.5	3.469 × 10^3^	4.365 × 10^3^	5.217 × 10^3^	1.131 × 10^3^
0.6	5.265 × 10^3^	6.244 × 10^3^	7.911 × 10^3^	1.341 × 10^3^
0.7	8.023 × 10^3^	8.045 × 10^3^	1.207 × 10^4^	1.528 × 10^3^
0.8	1.398 × 10^4^	9.724 × 10^3^	2.104 × 10^4^	1.682 × 10^3^
0.9	1.634 × 10^4^	1.118 × 10^4^	2.459 × 10^4^	1.871 × 10^4^
1.0	0	0	0	0

**Table 2:** The partial molar entropies of Bi and Sn at 903 K.

	Temperature 903 K	
*X*_*Bi*_	$∆{\bar{S}}_{Bi}$ [J/mol]	$∆{\bar{S}}_{Sn}$ [J/mol]
0.0	27.227	20.721
0.1	23.304	18.631
0.2	14.194	16.964
0.3	10.540	15.047
0.4	8.671	13.000
0.5	7.354	10.975
0.6	6.335	8.831
0.7	5.154	6.696
0.8	3.498	4.524
0.9	1.743	2.245
1.0	0	0

**Table 6:** XRF-EDP Test Report for Bi25Sn75 alloy sample.

Sample Name 1—Bi25Sn75 Description Method—TurboQuant-Alloys					
Z	Symbol	Element	Norm. Int.	Concentration	Abs. Error
13	Al	Aluminum	61.6353	0.0775%	0.0014%
15	P	Phosphorus	285.3130	0.08956%	0.00050%
20	Ca	Calcium	3071.7985	0.08837%	0.0010%
28	Ni	Nickel	100.9788	0.0527%	0.0032%
29	Cu	Copper	154.5501	0.0829%	0.0032%
50	Sn	Tin	30,350.5791	75.0850%	0.06%
72	Hf	Hafnium	105.3269	0.0157%	0.0084%
73	Ta	Tantalum	35.1758	0.0153%	0.0024%
74	W	Tungsten	63.0604	0.0623%	0.0031%
79	Au	Gold	534.9385	0.02587%	0.0054%
83	Bi	Bismuth	41,692.0316	24.317%	0.003%
		Other		0.0178%	
		Sum		99.93%	

**Table 7:** XRF-EDP Test Report for Bi50Sn50 alloy sample.

Sample Name 2—Bi50Sn50 Description Method—TurboQuant-Alloys					
Z	Symbol	Element	Norm. Int.	Concentration	Abs. Error
13	Al	Aluminum	88.0285	0.01747%	0.0032%
15	P	Phosphorus	583.8644	0.02200%	0.0010%
20	Ca	Calcium	1977.1571	0.08571%	0.0013%
28	Ni	Nickel	84.0540	0.0540%	0.0074%
29	Cu	Copper	185.2806	0.0354%	0.0076%
30	Zn	Zinc	24.0341	0.01265%	0.00094%
40	Zr	Zirconium	358.2148	0.05063%	0.0018%
47	Ag	Silver	50.5271	0.0137%	0.031%
50	Sn	Tin	18,162.4573	50.062%	0.05%
51	Sb	Antimony	26.0649	0.1180%	0.0027%
72	Hf	Hafnium	89.0883	0.0183%	0.021%
74	W	Tungsten	76.8009	0.1045%	0.0080%
78	Pt	Platinum	2567.5260	0.034%	0.073%
81	Tl	Thallium	845.4254	0.0402%	0.0062%
82	Pb	Lead	506.6176	0.0273%	0.0062%
83	Bi	Bismuth	97,237.1198	49.035%	0.01%
		Other		0.1768%	
		Sum		99.91%	

**Table 8:** XRF-EDP Test Report for Bi75Sn25 alloy sample.

Sample Name 3—Bi75Sn25 Description Method—TurboQuant-Alloys					
Z	Symbol	Element	Norm. Int.	Concentration	Abs. Error
13	Al	Aluminum	145.4822	0.0531%	0.0094%
20	Ca	Calcium	762.9267	0.06833%	0.0019%
28	Ni	Nickel	98.3900	0.0110%	0.019%
29	Cu	Copper	101.9181	0.0122%	0.018%
30	Zn	Zinc	39.4357	0.0354%	0.0025%
48	Cd	Cadmium	4.1658	0.0472%	0.0062%
50	Sn	Tin	5420.5119	24.9102%	0.05%
51	Sb	Antimony	13.5239	0.0119%	0.0035%
52	Te	Tellurium	8.0419	0.01203%	0.00058%
83	Bi	Bismuth	163,456.8712	74.7006%	0.02%
		Other		0.0918%	
		Sum		99.95%	

## Data Availability

Data are contained within the article.
